# Supplementary material for: The interactome of intact mitochondria by cross-linking mass spectrometry provides evidence for coexisting respiratory supercomplexes
Source: Mol Cell Proteomics. 2017 Dec 8;17(2):216–32. doi: 10.1074/mcp.RA117.000470 (PMC5795388; doi:10.1074/mcp.RA117.000470)
Supplement: Supplemental Data [file supp_RA117.000470_133922_0_supp_23978_fzffwf.zip › spectra_annotation/mito_DR_spectra_annotation/134-1-4-1-6-1.pdf]

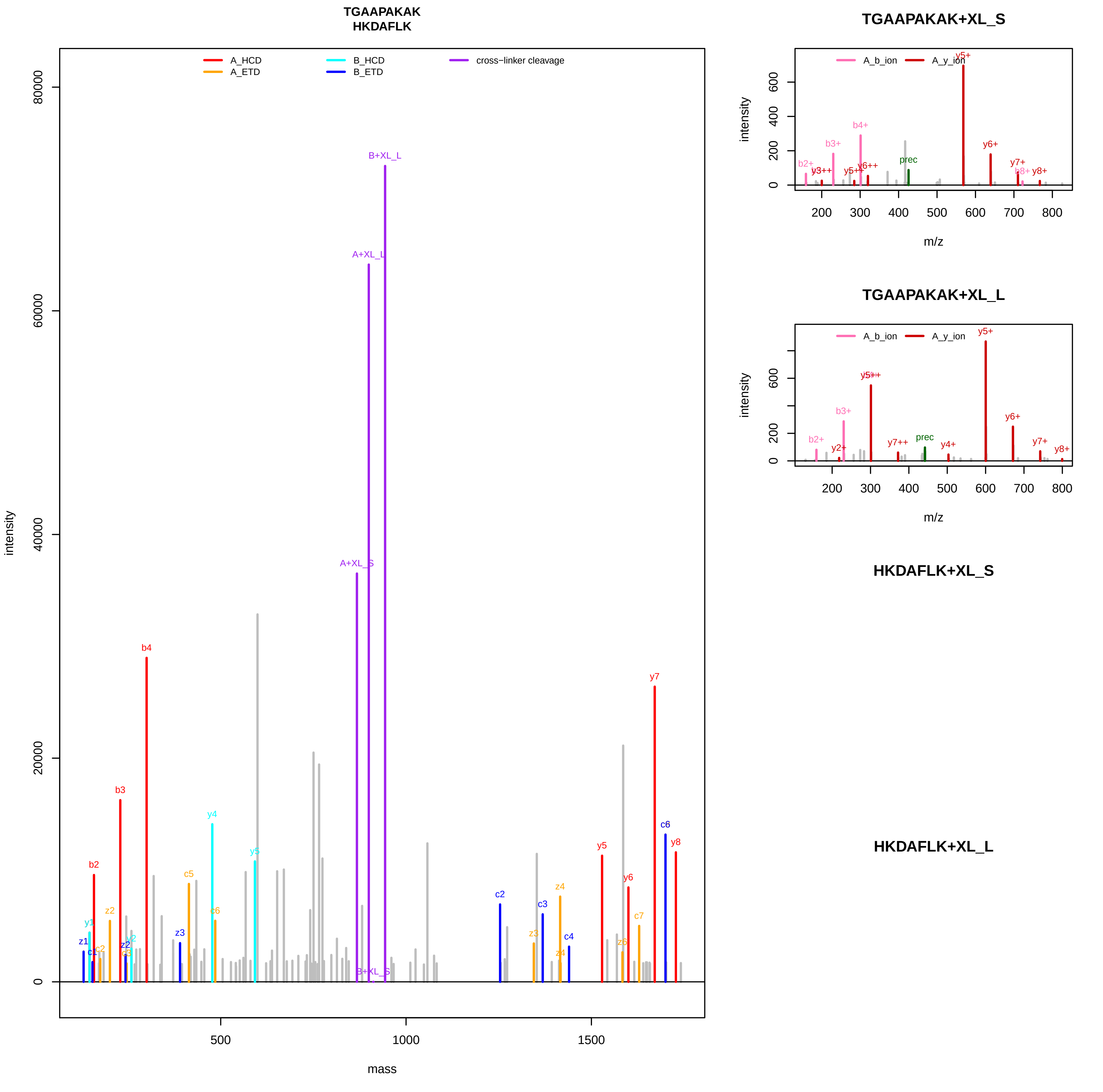

TGAAPAKAK
HKDAFLK
A_HCD
A_ETD
B_HCD
B_ETD
cross−linker cleavage
intensity
mass
0
20000
40000
60000
80000
500
1000
1500
B+XL_L
A+XL_L
A+XL_S
B+XL_S
b2
b3
b4
y1
y2
y4
y5
y6
y7
y8
z1
z2
z3
z4
z6
c1
c2
c3
c4
c5
c6
c7
TGAAPAKAK+XL_S
A_b_ion
A_y_ion
intensity
m/z
b2+
b3+
b4+
b8+
y3++
y5++
y6++
y5+
y6+
y7+
y8+
prec
TGAAPAKAK+XL_L
b2+
b3+
y2+
y5++
y7++
y4+
y5+
y6+
y7+
y8+
prec
HKDAFLK+XL_S
HKDAFLK+XL_L
